# Derivative Technology of DNA Barcoding (Nucleotide Signature and SNP Double Peak Methods) Detects Adulterants and Substitution in Chinese Patent Medicines

**DOI:** 10.1038/s41598-017-05892-y

**Published:** 2017-07-19

**Authors:** Zitong Gao, Yang Liu, Xiaoyue Wang, Jingyuan Song, Shilin Chen, Subramanyam Ragupathy, Jianping Han, Steven G. Newmaster

**Affiliations:** 1 Institute of Medicinal Plant Development, Chinese Academy of Medical Sciences & Peking Union Medical College, Beijing, 100193 China; 2 Institute of Chinese Materia Medica, China Academy of Chinese Medical Sciences, Beijing, 100700 P.R. China; 3 NHP Research Alliance, University of Guelph, Ontario, N1G2W1 Canada

## Abstract

Lonicerae japonicae Flos has been used to produce hundred kinds of Chinese patent medicines (CPMs) in China. Economically motivated adulterants have been documented, leading to market instability and a decline in consumer confidence. ITS2 has been used to identify raw medicinal materials, but it’s not suitable for the identification of botanical extracts and complex CPMs. Therefore, a short barcode for the identification of processed CPMs would be profitable. A 34 bp nucleotide signature (5′ CTAGCGGTGGTCGTACGATAGCCAATGCATGAGT 3′) was developed derived from ITS2 region of Eucommiae Folium based on unique motifs. Mixtures of powdered Lonicerae japonicae Flos and Lonicerae Flos resulted in double peaks at the expected SNP (Single Nucleotide Polymorphisms) positions, of which the height of the peaks were roughly indicative of the species’ ratio in the mixed powder. Subsequently we tested 20 extracts and 47 CPMs labelled as containing some species of *Lonicera*. The results revealed only 17% of the extracts and 22% of the CPMs were authentic, others exist substitution or adulterant; 7% were shown to contain both of two adulterants Eucommiae Folium and Lonicerae Flos. The methods developed in this study will widely broaden the application of DNA barcode in quality assurance of natural health products.

## Introduction

Adulteration and counterfeiting of medicinal plant products is a global problem^1, 2^, especially in developing areas such as Africa, Latin America, and Asia^3, 4^. The WHO (World Health Organization) estimated that as many as 30% of medicines sold in some Asian areas are adulterated^4–6^, and the occurrence of incorrect species as medicinal herb substitutes in Brazilian markets may be as high as 71%^7^. Even in developed countries, illegal supply chains aiding the diffusion of counterfeit drugs also exist^2^. Adulterated and counterfeit plant medicines are persistent in commercial markets, of which online businesses present considerable threats to both consumers’ health and a social security issues^8^. A previous study from our group showed that out of 1260 samples from 7 primary TCM markets in China, approximately 4.2% were identified as adulterants^9^. According to another study, only 40% of Rhodiola products on the market were authentic^10^. In China, there are many Chinese patent medicines (CPMs) with complex recipes. Decoction, extracts or CPMs made by spurious medicinal materials could undoubtedly be a considerable hazard to consumer health. Currently, a great number of herbal medicines are exported aboard in the form of extracts or CPMs. Therefore, more attention should be given to safety control of these products.

Lonicerae japonicae Flos (Jinyinhua) derived from *L*. j*aponica* is one of the most commonly used traditional Chinese medicines and is widely planted in China, Japan, Korea and Southeast Asia^11^ for the extraction of the main active medicinal components with high levels: chlorogenic acid (CGA)^12, 13^. Lonicerae japonicae Flos can be widely-ranged used in over hundreds of CPMs and natural health products (NHP) in the light of the Chinese Pharmacopeia (2015) or some sort of folk medicines, indicating the hugely demanded quantity and a considerable risk of adulteration or counterfeiting. There are claims in the industry that the high quality of Lonicerae japonicae Flos, rich in chlorogenic acid (CGA), are often adulterated or counterfeited for profit with Eucommiae Folium (*E. ulmoides Oliv;* Duzhongye) or Lonicerae Flos (Shanyinhua). Eucommiae Folium is derived from a completely different genera, and Lonicerae Flos can be derived from 4 different species including *L. marantha* (syn. *L. fulvotomentosa*)*, L. confusa, L. hypoglauca*, and *L. macranthoides*. Lonicerae Flos is much cheaper than Lonicerae japonicae Flos^14^. According to the theory of traditional Chinese medicine characteristics, Eucommiae Folium and Lonicerae japonicae Flos have totally different pharmacology, so the two herbal materials should be clearly distinguished. Microscopy is a primary method of identification in CPMs, but the unique microscopic characteristics are undetectable when herbs are ground into ultrafine powders with particle diameters of less than 10 microns. Additionally, it is hard to identify *Lonicera* species in extracts or in CPMs simply through HPLC using CGA as a marker. Based on the methods in the Chinese Pharmacopeia (2015), it is impossible detect Eucommiae Folium or Lonicerae Flos adulterants in extracts or CPMs labelled as Lonicerae japonicae Flos.

DNA barcoding has been used successfully to identify botanical species of medicinal plants^15–17^. The ITS2 (internal transcribed spacer 2), is a well-used DNA barcode region, exhibiting a 91% PCR recovery efficiency and possessing both high inter-species divergence and intra-species conservation^16, 18^. Hou *et al*. used ITS2 to identify Lonicerae japonicae Flos from Lonicerae Flos^19^, but this method is not feasible for extracts and CPMs where the DNA is heavily degraded into fragments that are smaller than what has been defined as a plant barcode region: >260 bp in ITS2 and >500 bp in *rbc* *L*. As almost every CPMs is prescription of complexity, merely one universal primer pair for ITS2 or any other barcode amplification for dozens of herbal products’ identification may not be a wise choice. Lo *et al*. demonstrated that 121 bp DNA fragments cannot be amplified in processed samples, whereas an 88 bp fragment was obtained successfully after the TCM materials had been boiled for 120 min^20^. Tiina Sarkinen also concluded that the PCR success rate had strong correlation with the amplicon size in which shorter fragments had a higher success rate^21^. DNA barcoding is somewhat antiquated as it is by a definition limited to the use of specific DNA barcode regions using Sanger sequencing, followed by species assignments within the barcode of life library, which has limited coverage for medicinal plant species and very poor estimates of intraspecific variation. This presents considerable impediments to the commercial use because the defined plant barcode regions are known to have low PCR and sequence success rates for processed samples^4, 22^. This is partly due to fact that DNA barcodes are too long for processed materials. Furthermore the proposed plant DNA barcode regions may not be useful to differentiate closely related medicinal species^23^.

We propose one derivative technology for testing commercial NHPs that build on the foundation of DNA barcoding. One approach is to utilize a “nucleotide signature” that is unique to the target species but much shorter than a common DNA barcode sequence. Our research presented here is focused on developing a contemporary molecular diagnostic tool for testing authentic Lonicerae japonicae Flos *(L. japonica* Thunb.; Jinyinhua) ingredients in NHPs. The goal of this study was to develop a short nucleotide signature for *E. ulmoides* and a SNP double peak detection method for Lonicerae Flos to distinguish the adulterant in the *Lonicera* extracts and CPMs. We validated these methods in detecting authentic species of Lonicerae japonicae Flos *(L. japonica* Thunb.; Jinyinhua) and targeted adulterant species including Eucommiae Folium (*E. ulmoides* Oliv.) and Lonicerae Flos (4 *Lonicera* spp.) by testing commercial market samples including 1) 20 extracts and 2) 47 CPMs.

## Results

### Nucleotide Signature Development and Detection for Ingredient Substitution

**Figure 1:**
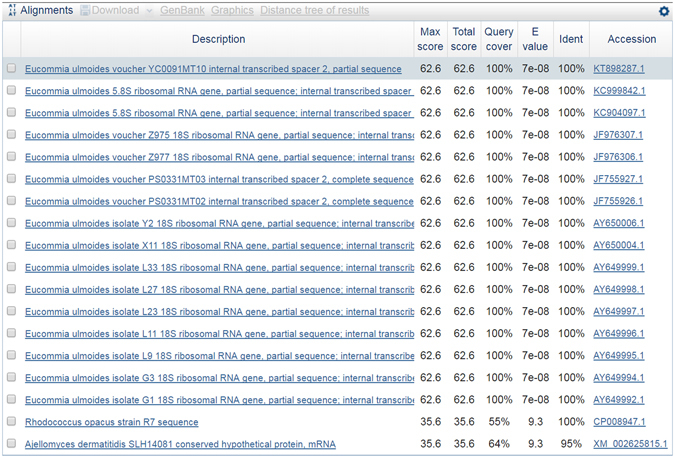
The alignment of the 34-nucleotide conserved region.

**Figure 2:**
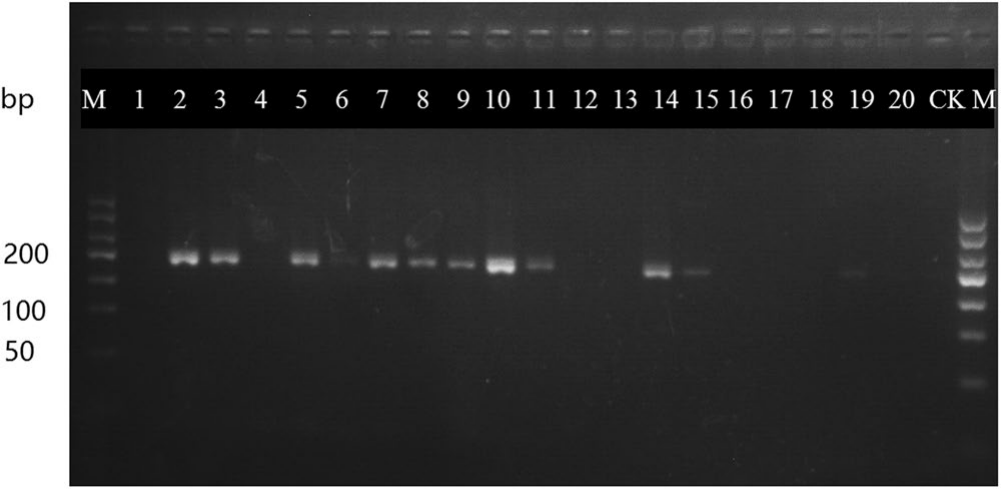
Specific primer pair DZF1/R1 amplification from extractives. Note: Except for the lanes marked M (marker) and CK (negative control), the remaining 20 lanes contain PCR products from 1 to 20 were Lonicerae japonicae Flos extracts amplified by DZF1/R1. All the order is the same as Table 1, and 2, 3, 5, 7–11, 14, 15 were represented the result that Eucommiae Folium can be detected through specific primer pair DZF1/R1.

We successfully developed a nucleotide signature and specific primer pairs for *E. ulmoides*. The success rate of the PCR amplification of the 420 bp ITS2 sequence by the universal primer pair 2 F/3R (More details about primer sequences in Table S2) from 24 Eucommiae Folium samples was 100%. There was no intraspecific variation detected when assessing the alignment of all the ITS2 sequences for all the populations Eucommiae Folium. We did not need to assess closely related species, as *E. ulmoides* is a single species in the *Eucommiaceae* family. According to the result of this alignment, one completely conserved 34 bp nucleotide signature (5′ CTAGCGGTGGTCGTACGATAGCCAATGCATGAGT 3′) from *E. ulmoides* was identified. BLAST results (Fig. 1) of this nucleotide signature demonstrated that this motif is unique to Eucommiae Folium and can be used to differentiate Eucommiae Folium from all the other species present in the NCBI database or BOLD database. In order to determine if this nucleotide signature detectable in processed NHP extracts, we tested 20 commercial extracts of Lonicerae japonicae Flos. A specific primer pair (DZF1/DZR1) (More details about primer sequences in Table S2) was developed to amplify the target fragments of *E. ulmoides* of which the length of the amplicon was *c*. 210 bp. The PCR products from this amplicon which were of the appropriate size were obtained and visualized on a gel (Fig. 2). The nucleotide signature was then sequenced to ensure that the identity of the amplified region from the extracts.

### Verification of the Nucleotide Signature Method Using NHP Extracts and CPMs

The nucleotide signature method successfully detected authentic and adulterated products in NHP extracts and CPMs. We used the species-specific primer pairs DZF1/DZR1 to amplify the nucleotide signature of Eucommiae Folium from the extracts and CPMs of Lonicerae japonicae Flos. The results showed that most targets could be amplified successfully, with exception of 2 extracts and 4 CPMs, which did not produce PCR products. A total of 18 batches of Lonicerae japonicae Flos extracts (TQWS1-S18) and 2 batches of Lonicerae Flos extracts (TQWS19-S20) were analysed (Table 1). Substitution with Eucommiae Folium was detected in 10/18 extracts and 5/44 CPMs, including Lingyangganmao soft capsules (ZCY03), Yinhuang soft capsules (ZCY04), Liyanjiedu particle (ZCY15), Jinsangkaiyin pills (ZCY22) and Xiaoerresuqing particles (ZCY34) (Table 2).

**Table 1:** Results of *Lonicerae japonicae* species extracts sequences analysis.

No.	Extracts Name	Purchase place	Sequences analysis results		
			Lonicerae japonicae Flos	Lonicerae Flos	Eucommiae Folium
TQWS1	Lonicerae japonicae Flos	Dalian, Liaoning	+	+	−
TQWS2	Lonicerae japonicae Flos	Dalian, Liaoning	+	++	+
TQWS3	Lonicerae japonicae Flos	Dalian, Liaoning	+	++	+
TQWS4	Lonicerae japonicae Flos	Dalian, Liaoning	+	++	−
TQWS5	Lonicerae japonicae Flos	Dalian, Liaoning	+	−	+
TQWS6	Lonicerae japonicae Flos	Dalian, Liaoning	+	−	−
TQWS7	Lonicerae japonicae Flos	Dalian, Liaoning	+	++	+
TQWS8	Lonicerae japonicae Flos	Dalian, Liaoning	+	++	+
TQWS9	Lonicerae japonicae Flos	Heze, Shandong	++	+	+
TQWS10	Lonicerae japonicae Flos	Changsha, Hunan	−	+	+
TQWS11	Lonicerae japonicae Flos	Changsha, Hunan	−	+	+
TQWS12	Lonicerae japonicae Flos	Xi’an Shaanxi	−	−	−
TQWS13	Lonicerae japonicae Flos	Xi’an Shaanxi	+	++	−
TQWS14	Lonicerae japonicae Flos	Xi’an, Shaanxi	++	+	+
TQWS15	Lonicerae japonicae Flos	Deyang, Sichuan	++	+	+
TQWS16	Lonicerae japonicae Flos	Chengdu, Sichuan	−	−	−
TQWS17	Lonicerae japonicae Flos	Chengdu, Sichuan	−	+	−
TQWS18	Lonicerae japonicae Flos	Chengdu, Sichuan	−	+	−
TQWS19	Lonicerae Flos	Chengdu, Sichuan	−	+	−
TQWS20	Lonicerae Flos	Chengdu, Sichuan	−	+	−

“+” means contain the component “−” means without the component. “++” means contain more quantity

**Table 2:** Results of Chinese Patent Medicines’ sequences analysis.

**No**.	**Samples**	**Listed species**	**Sequences analysis results**		
			Lonicerae japonicae Flos	Lonicerae Flos	Eucommiae Folium
ZCY01	Qingkailing pills	Lonicerae japonicae Flos	−	+	−
ZCY02	Lingyangqingfei particles	Lonicerae japonicae Flos	+	+	−
ZCY03	Lingyangganmaosoft capsules	Lonicerae japonicae Flos	−	+	+
ZCY04	Yinhuang soft capsules	Lonicerae japonicae Flos extract	++	+	+
ZCY05	Shuanghuanglian particles	Lonicerae japonicae Flose	+	+	−
ZCY06	Xiaoerjiebiao particles (first batch)	Lonicerae japonicae Flos	+	+	−
ZCY07	Ganmaozhikeparticles	Lonicerae Flos	+	+	−
ZCY08	Zhizijinhua pills (first batch)	Lonicerae japonicae Flos	−	+	−
ZCY09	Xiaoeryanbian particles	Lonicerae japonicae Flos	+	+	−
ZCY10	Jinqijiangtang tablets	Lonicerae japonicae Flos	+	−	−
ZCY11	Fufangyuxingcao tablets	Lonicerae japonicae Flos	−	−	−
ZCY12	Shouwu pills	Lonicerae japonicae Flos	−	+	−
ZCY13	Yinqiaojieduparticles	Lonicerae japonicae Flos	+	+	−
ZCY14	Weicyinqiao tablets	Lonicerae Flos	++	+	−
ZCY15	Liyanjiedu particles	Lonicerae japonicae Flos	++	+	+
ZCY16	Qingguo tablets	Lonicerae japonicae Flos	++	+	−
ZCY17	Kanggan particles	Lonicerae japonicae Flos	+	+	−
ZCY18	Xiaoyin capsules	Lonicerae japonicae Flos	+	+	−
ZCY19	Lianhuaqingwen capsules	Lonicerae japonicae Flos	+	−	−
ZCY20	Yinzhihuang particles (first batch)	Lonicerae japonicae Flos extract	+	−	−
ZCY21	Yinqiaosan	Lonicerae japonicae Flos	+	+	−
ZCY22	Jinsangkaiyin pills	Lonicerae japonicae Flos	+	−	+
ZCY23	Jinsangsanjie pills	Lonicerae japonicae Flos	+	−	−
ZCY24	Fufangjinyinhua particles (first batch)	Lonicerae japonicae Flos	+	+	−
ZCY25	Fufangganmaoling particles	Lonicerae japonicae Flos	+	−	−
ZCY26	Yinzhihuang particles (second batch)	Lonicerae japonicae Flos extract	−	−	−
ZCY27	Kouyanqing particles	Lonicerae Flos	+	+	−
ZCY28	Qingrejiedu capsules	Lonicerae japonicae Flos	−	+	−
ZCY29	Xiaocuo pills	Lonicerae japonicae Flos	−	−	−
ZCY30	Shuanghuanglian capsules	Lonicerae japonicae Flos	+	++	−
ZCY31	Biyuan tables	Lonicerae japonicae Flos	+	+	−
ZCY32	Qingkailing capsules	Lonicerae japonicae Flos	+	−	−
ZCY33	Yinhuang particles (first batch)	Lonicerae japonicae Flos extract	++	+	−
ZCY34	Xiaoerresuqing particles	Lonicerae japonicae Flos	+	−	+
ZCY35	Lingyangqingfei pills (first batch)	Lonicerae japonicae Flos	−	+	−
ZCY36	Lingyangqingfei pills (second batch)	Lonicerae japonicae Flos	++	+	−
ZCY37	Zhizijinhua pills (second batch)	Lonicerae japonicae Flos	−	+	−
ZCY38	Zhizijinhua pills (third batch)	Lonicerae japonicae Flos	+	+	−
ZCY39	Zhizijinhua pills (forth batch)	Lonicerae japonicae Flos	−	+	−
ZCY40	Lingqiaojiedu pills	Lonicerae japonicae Flos	+	−	−
ZCY41	Fufangjinyinhua particles (second batch)	Lonicerae japonicae Flos	++	+	−
ZCY42	Fufangjinyinhua paticles (third batch)	Lonicerae japonicae Flos	+	−	−
ZCY43	Yinhuang particles (second batch)	Lonicerae japonicae Flos extract	+	++	−
ZCY44	Yinzhihuang paricles (third batch)	Lonicerae japonicae Flos extract	++	+	−
ZCY45	Yinqiaoshangfeng capsules	Lonicerae Flos	−	+	−
ZCY46	Yinzhihuang soft capsule	Lonicerae jponicae Flos extract	+	−	−
ZCY47	Xiaoerjiebiao particles (second batch)	Lonicerae japonicae	+	++	−

+Present; -Absent. ++ Present in greater quantity.

### Development and Validation of Double Peaks Quantification Method Based on SNP Sites

**Figure 3:**
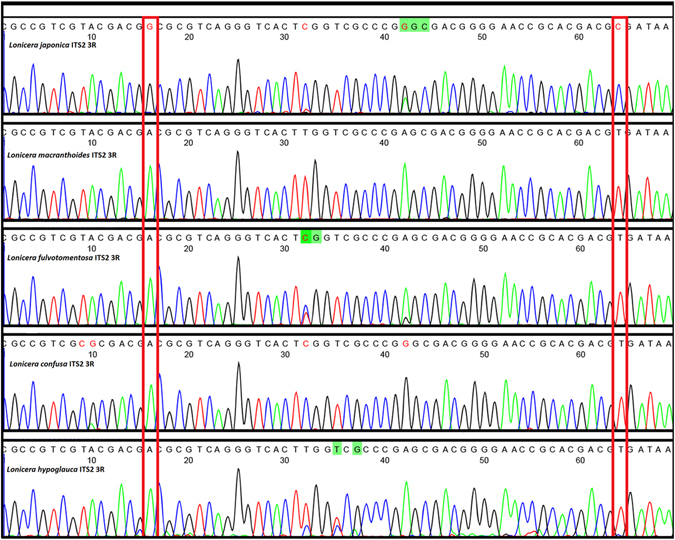
SNP peaks of *Lonicera japonica* and four originals of Lonicera Flos. Note: The Lonicera japonica and four species of Lonicera were amplified by ITS2F/3R, different bases were represented by different height of peaks and four different colours, two SNP sites were framed in this peak profile, respectively.

Two Single-nucleotide polymorphisms (SNPs) were identified for use in the double peak analysis *Lonicera* spp. used in NHPs. We evaluated 52 samples of *Lonicera*, *L. macranthoides, L. macrantha, L. confuse* and *L. hypoglauca* from all over China in which we amplified the ITS2 sequence using the universal primer pair 2 F/3R. The success rate of PCR amplification was 100%, and the size of ITS2 sequence was 420 bp. Sequence analysis of samples revealed 2 SNPs (16 bp and 64 bp) among the samples of Lonicerae japonicae Flos and Lonicera Flos. The SNP sites G and C specifically occurred in Lonicerae japonicae Flos, while A and T were present at the SNP positions in the four original species of Lonicerae Flos (Fig. 3). The specificity of the two SNPs was consistent among all the sequences obtained from Lonicerae materials.

**Figure 4:**
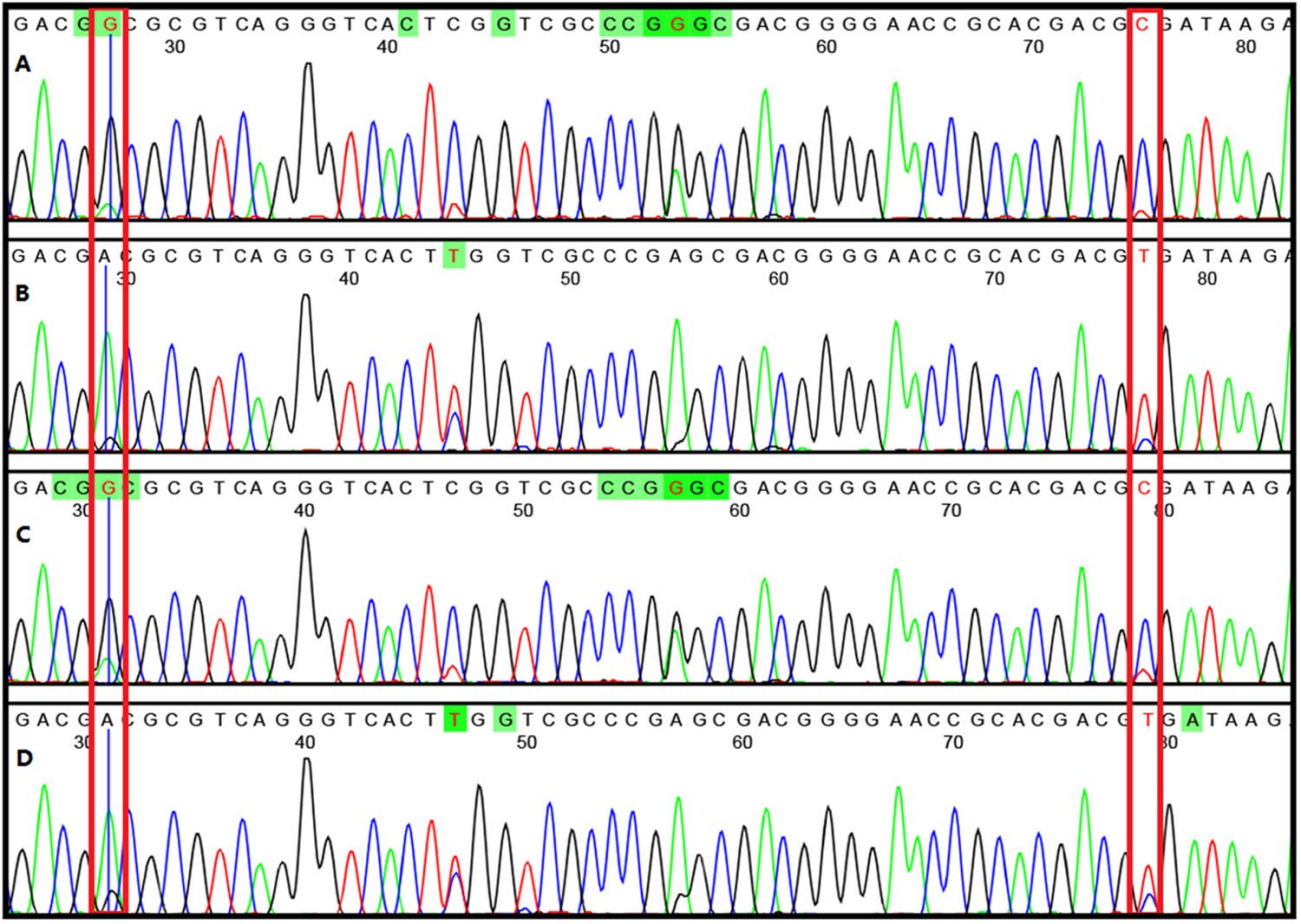
Double peaks of the artificial mixture of Lonicerae japonicae Flos and Lonicerae Flos from raw-plant powders. Note: Lonicerae japonicae Flos: Lonicerae Flos 15:1 (**A**) Lonicerae japonicae Flos: Lonicerae Flos 1:15 (**B**) Lonicerae japonicae Flos: Lonicerae Flos 4:1 (**C**) Lonicerae japonicae Flos: Lonicerae Flos 1:4 (**D**). Two SNP sites were framed in this peak profile, respectively.

Experimental mixtures of Lonicerae japonicae Flos and four different Lonicerae Flos were used to validate the quantitative estimates from the SNP double peak method. Powdered Lonicerae japonicae Flos - Lonicerae Flos were mixed in ratios (15:1, 4:1, 1:4, 1:15) that emulate adulteration. The trace data from PCR products of mixed powders with Lonicerae japonicae Flos-Lonicerae Flos ratios indicates that the relative amounts of substituted species can be detected by height of the two peaks at SNP positions (Fig. 4). In the impurity samples, Lonicerae Flos contamination rates can be detected from Lonicerae japonicae Flos as low as 6.2% (15:1) through the trace data (Fig. 4A). Double peaks could be clearly examined at SNP positions and the height of the peaks could indicate the ratio roughly, e.g. at the ratio of 15:1, the main peaks G/C attributed to Lonicerae japonicae Flos were much higher than the lower peaks A/T attributed to Lonicerae Flos, which means that the sample was adulterated with a little Lonicerae Flos. at the ratio of 1:15 (Fig. 4B), the main peaks A/T attributed to Lonicerae Flos were much higher than the lower peaks G/C attributed to Lonicerae japonicae Flos, which means that the sample was adulterated with a lot of Lonicerae Flos. Based on the results, we took the advantage of the SNP double peak methods for identification of the Chinese patent medicine.

The utility of the SNPs was tested on *Lonicera* NHP extracts. A specific primer pair (JYHF1/JYHR1) (More details about primer sequences in Table S2) was developed to amplify extracts based on the double peak method at the SNP sites. The length of the amplicon was *c*. 110 bp. 20 extracts were analysed of which two samples were not amplified. Diagnostic PCR products could be amplified successfully from 18 extracts of which double peaks were detected at the two SNP sites indicating that a sample contained both Lonicerae japonicae Flos and Lonicerae Flos. Further analysis of the peak trace at SNP sites indicated that 4 samples were substituted with Lonicerae Flos and 10 batches were adulterated with Lonicerae Flos. A comparison of the relative heights of the two peaks indicated that 6 batches of extracts contained more Lonicerae Flos than Lonicerae japonicae Flos. Only 1 batch of Lonicerae japonicae Flos and 2 batches of Lonicerae Flos were found to be authentic.

**Figure 5:**
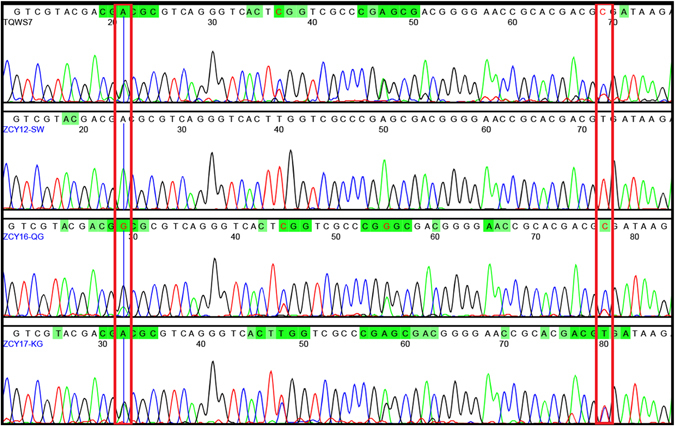
Double peaks at SNPs of adulterants of plant extracts and Chinese patent medicines. Note: One extracts (TQW 17) and three CPMs (ZCY12,16,17) were picked to demonstrate the SNP double peak methods. Two SNP sites were framed in this peak profile, respectively.

The utility of the SNPs was tested on Lonicerae Chinese patented medicine. In this study, all of the 47 CPMs are commonly used medicines, including 43 CPMs containing Lonicerae japonicae Flos and 4 batches containing Lonicerae Flos. A 110 bp fragment resulting from amplification with the specific primer pair JYHF1/JTHR1 was successfully observed for 44 samples (Table 2). The amplification efficiency was 93.62%. Double peak SNP were present and based on relative heights of the double peak, only 10 CPMs were authentic, 34 CPMs samples were substituted or adulterated. Quantitative estimates of mixtures are based on the relative heights of the double peaks (Fig. 5).

Double peaks are relative higher for Lonicerae japonicae Flos than that of Lonicerae Flos within the following samples (examples): Yinhuang soft capsules (ZCY04), Weicyinqiao tablets (ZCY14), Liyanjiedu particles (ZCY15), Qingguo tablets(ZCY16), Yinhuang particles (first batch)(ZCY33). Relative peaks are higher for the adulterant Lonicerae Flos in the following samples (examples): Lingyangqingfei particles (ZCY02), Shuanghuanglian particles (ZCY05), Yinqiaojiedu particles (ZCY13), Kanggan particles (ZCY17). Product substitution with Lonicerae Flos was found in the following samples (examples): Qingkailing pills (ZCY01), Zhizijinhua pills (first batch) (ZCY08), Shouwu pills (ZCY12). Substitution with large amounts of Lonicerae Flos, with only trace amounts of Lonicerae japonicae Flos detected within samples of Shuanghuanglian particles (ZCY30), Yinzhihuang particles (second batch) (ZCY43), Xiaoerjiebiao particles (second batch) (ZCY47). Three samples of the lesser-valued CPMs labelled as containing Lonicerae Flos actually contained the more valuable Lonicerae japonicae Flos; Moreover, 4 batches of CPMs contains Lonicerae Flos including Ganmaozhike particles, Weicyinqiao tablets, Kouyanqing particles and Yinqiaoshangfeng capsules.

There is considerable variation among batches for different groups of Chinese medicine. In order to compare the adulterants of the same CPMs from different batches, we analysed five different groups of CPMs including Yinzhihuang particles (three batches), Zhizijinhua pills (four batches), Lingyingqingfei pills (two batches), Fufangjinyinhua particles (three batches), xiaoerjiebiao particles (two batches) and Yinhuang particles (two batches). The first and the second batch of Fufangjinyinhua particles were substituted with Lonicerae Flos, but the second batch had relatively more Lonicerae japonicae Flos, and the third batch was authentic Lonicerae japonicae Flos. Among the four batches of Zhizijinhua pills, three of them were substituted with Lonicerae Flos, and one was substituted with Lonicerae Flos. The two batches of Yinhuang particles from different manufacturers were substituted with Lonicerae Flos, of which the first had more Lonicerae japonicae Flos and the second had more Lonicerae Flos.

### Nucleotide Signature and SNP Double Peak Methods combined for *Lonicera* NHP Quality Assurance

A combined analysis of the nucleotide signature and double peak methods revealed considerable adulteration of *Lonicera* NHPs. These results demonstrate the applicability of both primer pairs DZF1/DZR1 and JYHF1/R1 in the identification of Lonicerae japonicae Flos components in extracts and CPMs. We found that 9 batches of extracts consisted of both of two adulterants: Eucommiae Folium and Lonicerae Flos. Product substitution was detected in four samples, without any trace of Lonicerae japonicae Flos. Only one sample of Lonicerae japonicae Flos was authentic; unadulterated with neither Eucommiae Folium nor Lonicerae Flos detected. Two of the less expensive Lonicerae Flos extracts had no adulterants (Table 1). Among all of the 44 CPMs in this study, eight batches were substituted with Lonicerae Flos, 26 samples were found to be substituted, including three batches of CPMs (Lingyangganmao soft capsules (ZCY03),Yinhuang soft capsules (ZCY04), Liyanjiedu particles (ZCY15)), which contained both Eucommiae Folium and Lonicerae Flos, In addition, we detected six samples of extracts and three CPMs were found to be substituted with Lonicerae Flos more than Lonicerae japonicae Flos. Only ten CPMs were found to be authentic (Table 2).

## Discussion

### The Need for Molecular Diagnostics Tools for Quality Assurance of *Lonicera* NHPs

Lonicerae japonicae Flos is a widely used medicinal product in China, Japan, Korea, and Southeast of Asia^11^. The current quality assurance (QA) standard test for Lonicerae japonicae Flos products is utilizes analytical chemistry in detecting the content of CGA according to the current Chinese Pharmacopeia regulations^24^. There is great variation (5% to 95%) in the CGA content within Lonicerae japonicae Flos extracts in the market, which has become the basis for determining product prices. Compared to extracts, Chinese patent medicine (CPMs) and made up of complicated mixtures of herbs, which presents a considerable impediment for accurate analysis of CGA content. However, it is still used as a QA tool and detection index for identification of some CPMs containing Lonicerae japonicae Flos^25^. There are no publications that define the level variation in standard deviations associated with CGA measurements, nor statistics to assess the probability of false negatives or positive QA test. This is surprising given the amount of data that is available from routine QA testing of *Lonicera* NHPs. Managers of quality assurance in the NHP industry are searching for more accurate tools to assess *Lonicera* quality and assess the risk of adulteration in the supply chain.

Common adulterants pose considerable levels of uncertainty in the routine CGA estimates used in QA of *Lonicera* NHPs. Eucommiae Folium, Lonicerae japonicae Flos and Lonicerae Flos contain CGA and Eucommiae Folium has different pharmacological functions when mixed with Lonicerae japonicae Flos^26–29^. If these adulterants are present, then it is not possible to use CGA estimates as a detection method to distinguish between Lonicerae japonicae Flos and the common adulterants. Although there are many advanced chemical methods that provide appropriate analysis tools to adulteration in drugs and NHPs, the current standard methods in Chinese Pharmacopeia (2015) cannot determine if the samples are adulterated with Eucommiae Folium or Lonicerae Flos. This poses a problem for consumers, as they may not be getting the appropriate product and value that they have purchased. There is potential health risk to consumers as Lonicerae Flos can more easily lead to anaphylactic reactions than that of Lonicerae japonicae Flos^30^.

Molecular diagnostic tools provide an alternative method for NHP quality assurance and supply chain verification. The ITS2 sequence has been used to effectively identify the authenticity of raw herbal materials^31, 32^. The short length of ITS2 may have advantages over other DNA barcodes that are on average over 500 bp. However, the DNA barcode ITS2 may not be the best option for the identification of many mixed botanicals such as in CPMs, or processed extracts. This is because DNA degradation occurs during the manufacturing and processing steps, which leads to PCR amplification failure. Additionally, the complexity of the mixtures can lead to a mixed result and thus influence the level of accuracy. For CPMs like capsules, particles or pills, compressing and digesting are also compulsory parts of the production process according to the Chinese Pharmacopeia. The procedure of CPMs Shuanghuanglian particles production, for instance, includes boiling the Lonicerae japonicae Flos for 1.5 hours, filtrating in batches, and finally concentration of the product^33^. DNA degradation inevitably occurs during these multiple procedures and as a result, DNA sequences are always present as shorter fragments than is defined by DNA barcoding, even for the shorter region of ITS (260 bp). This may explain the failure of some PCR amplifications in many barcoding studies as the DNA template may have been too damaged to serve as a PCR template. Thus, mini-sequences (<120 bp) have become increasingly useful to enhance the success rate of obtaining DNA from NHPs^31^. According to research on *Gingko biloba* herbal dietary supplements, a designed mini-sequence approach has been used as a successful tool for detecting adulteration in the supply chain^34^. A mini-sequence based on a specific primer set may be unique to one species, which provides a unique molecular diagnostic tool for authenticating botanical ingredients^35^.

### Molecular Diagnostic Tools for Supply Chain Validation of Authentic *Lonicera* NHPs

There are many claims in the NHP industry that some manufactures deliberately add Eucommiae Folium and Lonicerae Flos to meet the standard of CGA indicated in the Chinese Pharmacopeia (2015). Unfortunately there is no analytical chemical method to detect these adulterants. Nucleotide signatures can be used to authenticate *Lonicera* NHPs (extracts and CPMs) and identify the common adulterant Eucommiae Folium. In previous researches, we successfully developed a nucleotide signature for American ginseng and Angelicae sinensis Radix^8, 36^. We successfully used nucleotide signatures to differentiate these two medicinal plants from common adulterants in powders and CPMs. This research provided a foundation for the work in the present study another short length (34 bp) nucleotide signature for the adulterant Eucommiae Folium in extracts or CPMs of Lonicerae japonicae Flos. *E. ulmoides* is a single species in the *Eucommiaceae* family that possess a unique nucleotide signature that is not a variable site within population; BLAST analysis with CBI GenBank and BOLD did not match to any other taxa. This unique nucleotide signature provides a quick tool that can be adapted to PCR technology. The food borne pathogen industry has utilized this approach as a commercial tool for quick screening of species within the supply chain. We suggest that this tool could be used for many NHP species including that of Ginseng noted above for quality assurance and supply chain validation that insures the authenticity of botanical ingredients.

### The SNP double peak detection method can be used to address issues of mixtures of adulterants

Adulteration with Lonicerae Flos is only one specific example of systemic problem in CPMs and many NHPs. SNP have been used extensively in molecular diagnostics as they are very useful for differentiating closely related species^37^. Chen *et al*. utilized the SNP double peak detection method to analyse mixtures of American ginseng and Panax ginseng by analysing the height of the double peak^38^. Our study provides further evidence that this method is useful for accurately detecting adulteration in experimental mixtures of more than two species. This required the use of two SNPs sites within the ITS2 region of *L. japonica*, *L. macranthoides, L.macrantha, L. confuse* and *L. hypoglauca*. We also found that the relative heights of the double peaks of mixtures of Lonicerae japonicae Flos and Lonicerae Flos, is a quantitative estimate of the relative amounts of adulteration. The SNP double peak method could be a quick method for quantitative adulterant detection in many other CPMs and NHPs.

Our study used molecular diagnostics to detect considerable adulteration in *Lonicera* NHPs. We found adulteration of commercial high value Lonicerae japonicae Flos with lesser-valued Lonicerae Flos in 33 CPMs and 12 extracts. Previous claims of adulteration in *Lonicera* NHPs by the industry are supported by our research and we have several plausible explanations. Firstly, the adulteration may occur early in the supply chain at the source of production on the farms. This is a complicated issue related to planting history and is not fraudulent adulteration, as the farmers may not understand the complexities of *Lonicera* nomenclature. This is particularly a problem when considering traditional common names for these Chinese medicines. Previously Lonicerae japonicae Flos or Lonicerae Flos were classified as the same herbal material^39^. Recently the Chinese Pharmacopeia (2015) has stipulated that Lonicerae Flos derived from *L. macrantha*, *L. confusa*, *L. hypoglauca* and *L. macranthoides* should be named “Shanyinghua”. Lonicerae japonicae Flos derived from *L. japonica* is called “Jingyinghua”. The change from synonymous to distinct names may have led to confusion among the farmers producing and labelling the raw materials in the supply chain. In our study, 3 batches of CPMs containing Lonicerae Flos were adulterated with the higher quality Lonicerae japonicae Flos, which indicates that the manufacturer could not distinguish the original materials, which resulted in a loss of potential revenue. Secondly, adulteration may be economically motivated. Lonicerae japonicae Flos is much more expensive than Lonicerae Flos, so some businesses may use Lonicerae Flos as a substitute to increase profits. Lastly, adulteration may occur because there are no tools to verify and validate authentic botanical ingredients for the quality control of *Lonicera* NHPs. It is a fact that the authentic and known adulterants have similar morphology (they look similar) and chemical composition. Our research provides a molecular diagnostic tool that addresses these plausible explanations for adulteration, and could be used for supply chain validation and possible regulation of *Lonicera* NHPs.

The regulation of *Lonicera* NHPs in China and globally needs to have careful consideration. Currently the Food and Drug Administration (FDA) in China considers it illegal when a CPM containing an unlisted species; this is a counterfeit CPMs. Although we provide molecular diagnostic tools for supply chain quality assurance, the use of this technology must be based on the need to differentiate *Lonicera* taxa. There is some research that suggests that there is no difference in medicinal efficacy^40, 41^. This might suggest that Lonicerae Flos could be merged into Lonicerae japonicae Flos. However, the Food and Drug Administration (FDA) in China stated that if manufacturers want to change the descriptions of *Lonicera* CPMs, there are should be an official inquiry and investigation of evidence to support such a change. Regardless of efficacy there is a perceived difference in the medicinal and consequent value of these different botanical ingredients and economically motivated adulteration is likely to occur. Our estimates (83% extracts and 78% CPMs) of *Lonicera* adulteration identify a considerable issue that must be addressed by all the stakeholders in the NHP industry including producers, suppliers, manufactures, distributors, regulators and perhaps most importantly the consumers.

## Materials and Methods

### Sampling of Study Material

A total of 76 herbal samples of *L. japonica*, *L. macranthoides*, *L. macrantha*, *L. confusa*, *L. hypoglauca* and *E. ulmoides* were collected as our biological reference material (BRM) library (Table S1). All the samples were authenticated at the species level by professor Yulin Lin (Institute of Medicinal Plant Development, Chinese Academy of Medical Sciences.) and voucher specimens were deposited in the herbarium of the Institute of Medicinal Plant Development. A total of 20 Lonicerae extracts that contained CGA levels ranging from 5–8% were collected from stores in Dalian city, Liaoning province, Xi’an city, Shaanxi province, Changsha city, Hunan province Heze city, Shandong province, Chengdu city and Deyang city, Sichuan province (Table 1). A total of 43 CPM containing Lonicerae japonicae Flos and 4 batches of CPM containing Lonicerae Flos described in the Chinese Pharmacopeia (2015) were purchased from drug stores in Beijing and online pharmacy stores (Table 2).

### DNA Extraction, Amplification, and Sequencing

Flower buds and leaves were sterilized with 75% ethanol, and approximately 20 mg of each sample was then ground by ball-milling (Retsch, Germany). DNA was extracted with the Plant Universal Genomic DNA Kit (Tiangen Biotech Beijing Co., China) according to the manufacturer’s instructions. Amplification of ITS2 sequence was performed by the universal primer pair ITS2 2 F/3R (2.5µmol/µL). The PCR mixture contained 2 µL template DNA, 2.5 µL PCR buffer (10×), 2 µL Mg^2+^(25 mmol/L), 2 µL dNTP mixture (2.5 mmol/L), 1.0 µL 2 F/3R (2.5µmol/L)^42^, and 1.0 U Taq DNA polymerase. PCR was run for a total of 40 cycles with the following temperatures and times: 94 °C for 30 s, 56 °C for 30 s, and 72 °C for 45 s, followed by an incubation at 72 °C for 10 min. After detection of PCR products by gel electrophoresis, PCR products were bidirectionally sequenced using the ABI 3730XL DNA sequencer (Applied Biosystems).

### Extracts and Chinese Patent Medicine

A total of 40–50 mg of extracts or Chinese patent medicine were placed in a tube and ground by ball-milling machine (Retsch, Germany). Six tubes were taken in parallel for each batch of extracts or CPMs. DNA was extracted with the Plant Universal Genomic DNA Kit (Tiangen Biotech Beijing Co., China) according to the manufacturer’s instructions. Finally, DNA from each batch of extracts or CPMs was combined in a single tube and concentrated. The degraded DNA was amplified using two primer pairs (DZF1/DZR1 and JYHF1/JYHR1) newly designed using Primer 6.0 software. Details of these primers are shown in Table S2. The PCR mixture contained 2 µL template DNA, 2.5 µL PCR buffer (10×), 2 µL Mg^2+^ (25 mmol/L), 2 µL dNTP mixture (2.5 mmol/L), 2.0 µL specific primers DZF1/DZR1 and JYHF1/JYHR1 (2.5µmol/L) separately, and 1.0 U Taq DNA polymerase. PCR was run for a total of 40 cycles at 94 °C for 30 s, 56 °C for 30 s, and 72 °C for 45 s, followed by incubation at 72 °C for 10 min. After the detection of PCR products by gel electrophoresis, the PCR products were directionally sequenced using the ABI 3730XL DNA sequencer (Applied Biosystems).

### Mixture of Lonicerae japonicae Flos and Lonicerae Flos Raw-Plant Powders

Lonicerae japonicae Flos and Lonicerae Flos were mixed at ratios of 15:1, 4:1, 1:4 and 1:15. Then, DNA was extracted from the mixed powder with the Plant Universal Genomic DNA Kit (Tiangen Biotech Beijing Co., China) according to the manufacturer’s instructions. Amplification of nucleotide signatures from single species and from the mixtures was performed using the specific primer pair JYHF1/JYHR1(2.5µmol/µL). PCR products were bidirectionally sequenced using the ABI 3730XL DNA sequencer (Applied Biosystems).

### Sequence Analysis

ITS2 sequences were assembled using CodonCode Aligner 3.7.1 (CodonCode Co., Germany). The low-quality sequence data were removed from both ends of the sequencing results. All ITS2 sequences from *E. ulmoides* and *Lonicera* were annotated and delimited using a hidden Markov Model (HMM)-based method to eliminate the 5.8 S and 28 S rDNA regions^43^. The haplotypes of every *E. ulmoides* and *Lonicera* species were selected by CodonCode Aligner. All the haplotypes were then aligned using MEGA 5 software^44^.

## Electronic supplementary material

Table S1

Table S2
