# Supplementary material for: Derivative Technology of DNA Barcoding (Nucleotide Signature and SNP Double Peak Methods) Detects Adulterants and Substitution in Chinese Patent Medicines
Source: Sci Rep. 2017 Jul 19;7:5858. doi: 10.1038/s41598-017-05892-y (PMC5517575; doi:10.1038/s41598-017-05892-y)
Supplement: Supplementary file 1 — Table S1 [file 41598_2017_5892_MOESM1_ESM.pdf]

Derivative Technology of DNA Barcoding (Nucleotide Signature and SNP Double Peak Methods) Detects Adulterants and Substitution in Chinese Patent Medicines

Zitong Gao1, Yang Liu1, Xiaoyue Wang1， Jingyuan Song1， Shilin Chen2, Subramanyam Ragupathy3， Jianping Han1*， Steven G Newmaster3 *

1Institute of Medicinal Plant Development, Chinese Academy of Medical Sciences & Peking Union Medical College, Beijing 100193, China;
2Institute of Chinese Materia Medica, China Academy of Chinese Medical Sciences, Beijing 100700, P.R. China;
3NHP Molecular Diagnostics R&D Lab, BIO, University of Guelph, Department of Integrative Biology, Ontario, Canada

Table S1 A list of total of 76 population samples

Sample No.	Latin Name of Original Species	Latin Name of Medicinal Materials	Collection Site	Sample Type	Identification Result
YC0014MT15	*Lonicera japonica*	Lonicerae japonicae Flos	Pingyi ,Shandong	Flower Bud	*Lonicera japonica*
YC0014MT16	*Lonicera japonica*	Lonicerae japonicae Flos	Pingyi ,Shandong	Flower Bud	*Lonicera japonica*
YC0014MT29	*Lonicera japonica*	Lonicerae japonicae Flos	Xixia, Henan	Flower Bud	*Lonicera japonica*
YC0014MT31	*Lonicera japonica*	Lonicerae japonicae Flos	Xixia, Henan	Flower Bud	*Lonicera japonica*
YC0014MT32	*Lonicera japonica*	Lonicerae japonicae Flos	Xixia, Henan	Flower Bud	*Lonicera japonica*
YC0014MT33	*Lonicera japonica*	Lonicerae japonicae Flos	Xixia, Henan	Flower Bud	*Lonicera japonica*
YC0014MT41	*Lonicera japonica*	Lonicerae japonicae Flos	Fengqiu, Henan	Flower Bud	*Lonicera japonica*
YC0014MT43	*Lonicera japonica*	Lonicerae japonicae Flos	Fengqiu, Henan	Flower Bud	*Lonicera japonica*
YC0014MT54	*Lonicera japonica*	Lonicerae japonicae Flos	Donghai, Jiangsu	Flower Bud	*Lonicera japonica*
YC0014MT55	*Lonicera japonica*	Lonicerae japonicae Flos	Donghai, Jiangsu	Flower Bud	*Lonicera japonica*
YC0014MT57	*Lonicera japonica*	Lonicerae japonicae Flos	Donghai, Jiangsu	Flower Bud	*Lonicera japonica*
YC0014MT58	*Lonicera japonica*	Lonicerae japonicae Flos	Donghai, Jiangsu	Flower Bud	*Lonicera japonica*
YC0014MT59	*Lonicera japonica*	Lonicerae japonicae Flos	Donghai, Jiangsu	Flower Bud	*Lonicera japonica*
YC0014MT60	*Lonicera japonica*	Lonicerae japonicae Flos	Pingyi, Shandong	Flower Bud	*Lonicera japonica*
YC0014MT62	*Lonicera japonica*	Lonicerae japonicae Flos	Pingyi, Shandong	Flower Bud	*Lonicera japonica*
YC0014MT63	*Lonicera japonica*	Lonicerae japonicae Flos	Pingyi, Shandong	Flower Bud	*Lonicera japonica*
YC0014MT81	*Lonicera japonica*	Lonicerae japonicae Flos	Liupanshui, Guizhou	Flower Bud	*Lonicera japonica*
YC0014MT98	*Lonicera japonica*	Lonicerae japonicae Flos	Zhoukou, Henan	Flower Bud	*Lonicera japonica*
YC0014MT101	*Lonicera japonica*	Lonicerae japonicae Flos	Shucheng, Anhui	Flower Bud	*Lonicera japonica*
YC0014MT104	*Lonicera japonica*	Lonicerae japonicae Flos	Huanggang, Hubei	Flower Bud	*Lonicera japonica*
YC0014MT108	*Lonicera japonica*	Lonicerae japonicae Flos	Julu, Hebei	Flower Bud	*Lonicera japonica*
YC0014MT109	*Lonicera japonica*	Lonicerae japonicae Flos	Julu, Hebei	Flower Bud	*Lonicera japonica*
YC0014MT110	*Lonicera japonica*	Lonicerae japonicae Flos	Julu, Hebei	Flower Bud	*Lonicera japonica*
YC0163MT04	*Lonicera macranthoide*	Lonicerae Flos	Banan, Chongqing	Flower Bud	*Lonicera macranthoide*
YC0163MT05	*Lonicera macranthoide*	Lonicerae Flos	Banan, Chongqing	Flower Bud	*Lonicera macranthoide*
YC0163MT06	*Lonicera macranthoide*	Lonicerae Flos	Banan, Chongqing	Flower Bud	*Lonicera macranthoide*
YC0163MT07	*Lonicera macranthoide*	Lonicerae Flos	Banan, Chongqing	Flower Bud	*Lonicera macranthoide*
YC0163MT08	*Lonicera macranthoide*	Lonicerae Flos	Banan, Chongqing	Flower Bud	*Lonicera macranthoide*
YC0163MT09	*Lonicera macranthoide*	Lonicerae Flos	Banan, Chongqing	Flower Bud	*Lonicera macranthoide*
YC0163MT10	*Lonicera macranthoide*	Lonicerae Flos	Banan, Chongqing	Flower Bud	*Lonicera macranthoide*
YC0163MT20	*Lonicera macranthoide*	Lonicerae Flos	Banan, Chongqing	Flower Bud	*Lonicera macranthoide*
YC0163MT21	*Lonicera macranthoide*	Lonicerae Flos	Longhui, Hunan	Flower Bud	*Lonicera macranthoide*
YC0163MT25	*Lonicera macranthoide*	Lonicerae Flos	Nanjiang, Sichuan	Flower Bud	*Lonicera macranthoide*

YC0163MT26	*Lonicera macranthoide*	Lonicerae Flos	Puer, Yunan	Flower Bud	*Lonicera macranthoide*
YC0164MT01	*Lonicera fulvotomentos*	Lonicerae Flos	Anlong,Guizhou	Flower Bud	*Lonicera fulvotomentos*
YC0164MT02	*Lonicera fulvotomentos*	Lonicerae Flos	Anlong,Guizhou	Flower Bud	*Lonicera fulvotomentos*
YC0164MT04	*Lonicera fulvotomentos*	Lonicerae Flos	Xingren,Guizhou	Flower Bud	*Lonicera fulvotomentos*
YC0164MT05	*Lonicera fulvotomentos*	Lonicerae Flos	Xingren,Guizhou	Flower Bud	*Lonicera fulvotomentos*
YC0164MT06	*Lonicera fulvotomentos*	Lonicerae Flos	Xingren,Guizhou	Flower Bud	*Lonicera fulvotomentos*
YC0164MT07	*Lonicera fulvotomentos*	Lonicerae Flos	Xingren,Guizhou	Flower Bud	*Lonicera fulvotomentos*
YC0164MT08	*Lonicera fulvotomentos*	Lonicerae Flos	Zhenfeng, Guizhou	Flower Bud	*Lonicera fulvotomentos*
YC0164MT09	*Lonicera fulvotomentos*	Lonicerae Flos	Zerong,Guizhou	Flower Bud	*Lonicera fulvotomentos*
YC0164MT11	*Lonicera fulvotomentos*	Lonicerae Flos	Xingyi, Guizhou	Flower Bud	*Lonicera fulvotomentos*
YC0164MT12	*Lonicera fulvotomentos*	Lonicerae Flos	Fengqiu, Henan	Flower Bud	*Lonicera fulvotomentos*
YC0164MT17	*Lonicera fulvotomentos*	Lonicerae Flos	Anlong,Guizhou	Flower Bud	*Lonicera fulvotomentos*
YC0164MT19	*Lonicera fulvotomentos*	Lonicerae Flos	Anlong,Guizhou	Flower Bud	*Lonicera fulvotomentos*
YC0165MT01	*Lonicera hypoglauca*	Lonicerae Flos	Nanning, Guangxi	Flower Bud	*Lonicera hypoglauca*
YC0165MT02	*Lonicera hypoglauca*	Lonicerae Flos	Hezhou,Guangxi	Flower Bud	*Lonicera hypoglauca*
YC0166MT01	*Lonicera confusa*	Lonicerae Flos	Nanning, Guangxi	Flower Bud	*Lonicera confusa*
YC0166MT04	*Lonicera confusa*	Lonicerae Flos	Shipan,Sichuan	Flower Bud	*Lonicera confusa*
YC0166MT06	*Lonicera confusa*	Lonicerae Flos	Shipan,Sichuan	Flower Bud	*Lonicera confusa*
YC0166MT07	*Lonicera confusa*	Lonicerae Flos	Jianyang, Sichuan	Flower Bud	*Lonicera confusa*
SBC20	*Eucommia ulmoides*	Eucommiae Folium	Implad	Leaves	*Eucommia ulmoides*
SBC21	*Eucommia ulmoides*	Eucommiae Folium	Implad	Leaves	*Eucommia ulmoides*
DZ7	*Eucommia ulmoides*	Eucommiae Folium	Implad	Leaves	*Eucommia ulmoides*
DZ12	*Eucommia ulmoides*	Eucommiae Folium	Implad	Leaves	*Eucommia ulmoides*
DZ13	*Eucommia ulmoides*	Eucommiae Folium	Implad	Leaves	*Eucommia ulmoides*
DZ14	*Eucommia ulmoides*	Eucommiae Folium	Implad	Leaves	*Eucommia ulmoides*
DZ1	*Eucommia ulmoides*	Eucommiae Folium	Lvye Company	Leaves	*Eucommia ulmoides*
DZ2	*Eucommia ulmoides*	Eucommiae Folium	Hebei Anguo Herb Market	Leaves	*Eucommia ulmoides*
X402	*Eucommia ulmoides*	Eucommiae Folium	Implad	Leaves	*Eucommia ulmoides*
X403	*Eucommia ulmoides*	Eucommiae Folium	Implad	Leaves	*Eucommia ulmoides*
YC0091MT16	*Eucommia ulmoides*	Eucommiae Folium	Nanjing Guangxi	Leaves	*Eucommia ulmoides*
YC0091MT17	*Eucommia ulmoides*	Eucommiae Folium	Nanjing Guangxi	Leaves	*Eucommia ulmoides*
YC0091MT18	*Eucommia ulmoides*	Eucommiae Folium	Nanjing Guangxi	Leaves	*Eucommia ulmoides*
YC0091MT19	*Eucommia ulmoides*	Eucommiae Folium	Nanjing Guangxi	Leaves	*Eucommia ulmoides*
YC0091MT20	*Eucommia ulmoides*	Eucommiae Folium	Nanjing Guangxi	Leaves	*Eucommia ulmoides*
YC0091MT21	*Eucommia ulmoides*	Eucommiae Folium	Nanjing Guangxi	Leaves	*Eucommia ulmoides*

YC0091MT22	*Eucommia ulmoides*	Eucommiae Folium	Nanjing Guangxi	Leaves	*Eucommia ulmoides*
YC0091MT23	*Eucommia ulmoides*	Eucommiae Folium	Nanjing Guangxi	Leaves	*Eucommia ulmoides*
YC0091MT24	*Eucommia ulmoides*	Eucommiae Folium	Nanjing Guangxi	Leaves	*Eucommia ulmoides*
YC0091MT25	*Eucommia ulmoides*	Eucommiae Folium	Nanjing Guangxi	Leaves	*Eucommia ulmoides*
YC0091MT26	*Eucommia ulmoides*	Eucommiae Folium	Nanjing Guangxi	Leaves	*Eucommia ulmoides*
YC0091MT27	*Eucommia ulmoides*	Eucommiae Folium	Nanjing Guangxi	Leaves	*Eucommia ulmoides*
YC0091MT39	*Eucommia ulmoides*	Eucommiae Folium	Chengkou Chongqing	Leaves	*Eucommia ulmoides*
YC0091MT40	*Eucommia ulmoides*	Eucommiae Folium	Chengkou Chongqing	Leaves	*Eucommia ulmoides*

Note: Implad refers to Institute of Medicinal Plant Development
