# Supplementary material for: Derivative Technology of DNA Barcoding (Nucleotide Signature and SNP Double Peak Methods) Detects Adulterants and Substitution in Chinese Patent Medicines
Source: Sci Rep. 2017 Jul 19;7:5858. doi: 10.1038/s41598-017-05892-y (PMC5517575; doi:10.1038/s41598-017-05892-y)
Supplement: Supplementary file 2 — Table S2 [file 41598_2017_5892_MOESM2_ESM.pdf]

Derivative Technology of DNA Barcoding (Nucleotide Signature and SNP Double Peak Methods) Detects Adulterants and Substitution in Chinese Patent Medicines

Zitong Gao1, Yang Liu1, Xiaoyue Wang1， Jingyuan Song1， Shilin Chen2, Subramanyam Ragupathy3， Jianping Han1*， Steven G Newmaster3 *

1Institute of Medicinal Plant Development, Chinese Academy of Medical Sciences & Peking Union Medical College, Beijing 100193, China;
2Institute of Chinese Materia Medica, China Academy of Chinese Medical Sciences, Beijing 100700, P.R. China;
3NHP Molecular Diagnostics R&D Lab, BIO, University of Guelph, Department of Integrative Biology, Ontario, Canada

Table S2 Sequences of primer pairs

Primer Pairs	Forward	Reverse
ITS2R/3F	ATTCACACCAAGTATCGCAT	ATTGTAGTCTGGAGAAGCGTC
DZF1/R1	GAGTCTTTGAACGCAAGTTG	GACGGCACGGATGCTTAA
JYHF1/R1	AGTGGTGGTCGTAACATTC	TCCTCCGCTTATTGATATGC
